# Genetic reprogramming with stem cells regenerates glomerular epithelial podocytes in Alport syndrome

**DOI:** 10.26508/lsa.202402664

**Published:** 2024-03-28

**Authors:** Valerie S LeBleu, Keizo Kanasaki, Sara Lovisa, Joseph L Alge, Jiha Kim, Yang Chen, Yingqi Teng, Behzad Gerami-Naini, Hikaru Sugimoto, Noritoshi Kato, Ignacio Revuelta, Nicole Grau, Jonathan P Sleeman, Gangadhar Taduri, Akane Kizu, Shahin Rafii, Konrad Hochedlinger, Susan E Quaggin, Raghu Kalluri

**Affiliations:** 1 Department of Cancer Biology, University of Texas MD Anderson Cancer Center, Houston, TX, USA; 2 Division of Matrix Biology, Beth Israel Deaconess Medical Center and Harvard Medical School, Boston, MA, USA; 3 Northwestern University Feinberg School of Medicine and Kellogg School of Management, Chicago, IL, USA; 4 Department of Medicine, Baylor College of Medicine , Houston, TX, USA; 5 Medical Faculty Mannheim, University of Heidelberg, Heidelberg, Germany; 6 Department of Radiation Oncology, Heidelberg University Hospital, Heidelberg, Germany; 7 Karlsruhe Institute of Technology (IBCS-BIP), Karlsruhe, Germany; 8 Department of Genetic Medicine and Ansary Stem Cell Institute, Weill Cornell Medical College, New York, NY, USA; 9 Massachusetts General Hospital, Boston, MA, USA; 10 Harvard Stem Cell Institute, Boston, MA, USA; 11 Northwestern University Feinberg School of Medicine & Feinberg Cardiovascular and Renal Research Institute, Chicago, IL, USA; 12 Harvard-MIT Division of Health Sciences and Technology, Boston, MA, USA; 13 Department of Bioengineering, Rice University, Houston, TX, USA; 14 Department of Molecular and Cellular Biology, Baylor College of Medicine , Houston, TX, USA

## Abstract

Glomerular filtration relies on the type IV collagen (ColIV) network of the glomerular basement membrane, namely, in the triple helical molecules containing the α3, α4, and α5 chains of ColIV. Loss of function mutations in the genes encoding these chains (*Col4a3*, *Col4a4*, and *Col4a5*) is associated with the loss of renal function observed in Alport syndrome (AS). Precise understanding of the cellular basis for the patho-mechanism remains unknown and a specific therapy for this disease does not currently exist. Here, we generated a novel allele for the conditional deletion of *Col4a3* in different glomerular cell types in mice. We found that podocytes specifically generate α3 chains in the developing glomerular basement membrane, and that its absence is sufficient to impair glomerular filtration as seen in AS. Next, we show that horizontal gene transfer, enhanced by TGFβ1 and using allogenic bone marrow–derived mesenchymal stem cells and induced pluripotent stem cells, rescues *Col4a3* expression and revive kidney function in Col4a3-deficient AS mice. Our proof-of-concept study supports that horizontal gene transfer such as cell fusion enables cell-based therapy in Alport syndrome.

## Abstract (precis)

Podocytes are the rate-limiting glomerular cells for type IV collagen production, and horizontal gene transfer or cell fusion with stem cells regenerates the renal parenchyma in Alport syndrome.

## Introduction

Alport syndrome (AS), the progressive hereditary kidney disease that is estimated to impact approximately 1 in 50,000 newborns, is associated with aberrant glomerular basement membrane (GBM) type IV collagen composition, and it remains a clinical challenge that ultimately requires renal transplant. GBM defects in AS arise from mutations in either of the three genes (*Col4a3*, *Col4a4*, or *Col4a5* genes) that encode for three chains of type IV collagen, respectively (Hudson et al, 2003; Cosgrove et al, 2007; LeBleu et al, 2010; Cosgrove & Liu, 2017; Boudko et al, 2022). Patho-mechanistic studies suggest that loss of one chain type can lead to loss of the other two chain types due to the obligate requirement for integration of the three α-chain types into the triple helical type IV collagen molecule (α3α4α5 triple helical protomers). Interestingly, loss of GBM type IV collagen α3, α4, or α5 chain leads to compensation by α1 and α2 chains (α1α2α1 triple helical protomers), but this does not confer the structural and functional integrity for proper glomerular filtration (Naylor et al, 2021). Non-targeted therapies for glomerular and interstitial renal diseases have been tested with some benefit in patients with Alport syndrome, but they failed to prevent renal failure (Katayama et al, 2014; Omachi & Miner, 2019; Chavez et al, 2022; Daga et al, 2022).

Previously, systemic infusion of unfractionated bone marrow–derived cells and embryonic stem cells were reported to restore the GBM type IV collagen composition in *Col4a3* knock out (Col4a3^KO^) mice (a mouse model for AS) with increased survival, improved renal function, and histopathological regeneration of glomeruli (Prodromidi et al, 2006; Sugimoto et al, 2006). These proof-of-concept studies raise the question as to the precise function of stem cells in the regeneration of the kidney glomeruli and their clinical translation potential (Heidet et al, 2003; Gross et al, 2009; LeBleu et al, 2009; Lin et al, 2014). Glomerular endothelial cells and podocytes are hypothesized to contribute to the α3α4α5 type IV collagen of the GBM (Abrahamson et al, 2009), but genetic evidence is still lacking. To address this issue directly, we generated a novel genetically engineered mouse that enables the conditional deletion of *Col4α3* in specific cell types to identify the origin of GBM type IV collagen. Using cell fate-mapping strategies, we designed experiments to define the underlying mechanism associated with stem cell–based rescue of renal failure in Col4a3^KO^ mice, utilizing allogenic bone marrow–derived mesenchymal stem cells (MSC) and induced pluripotent stem cells (IPS).

## Results

### Glomerular podocytes are rate-limiting producers of *Col4α3* in the GBM

**Figure 1.**
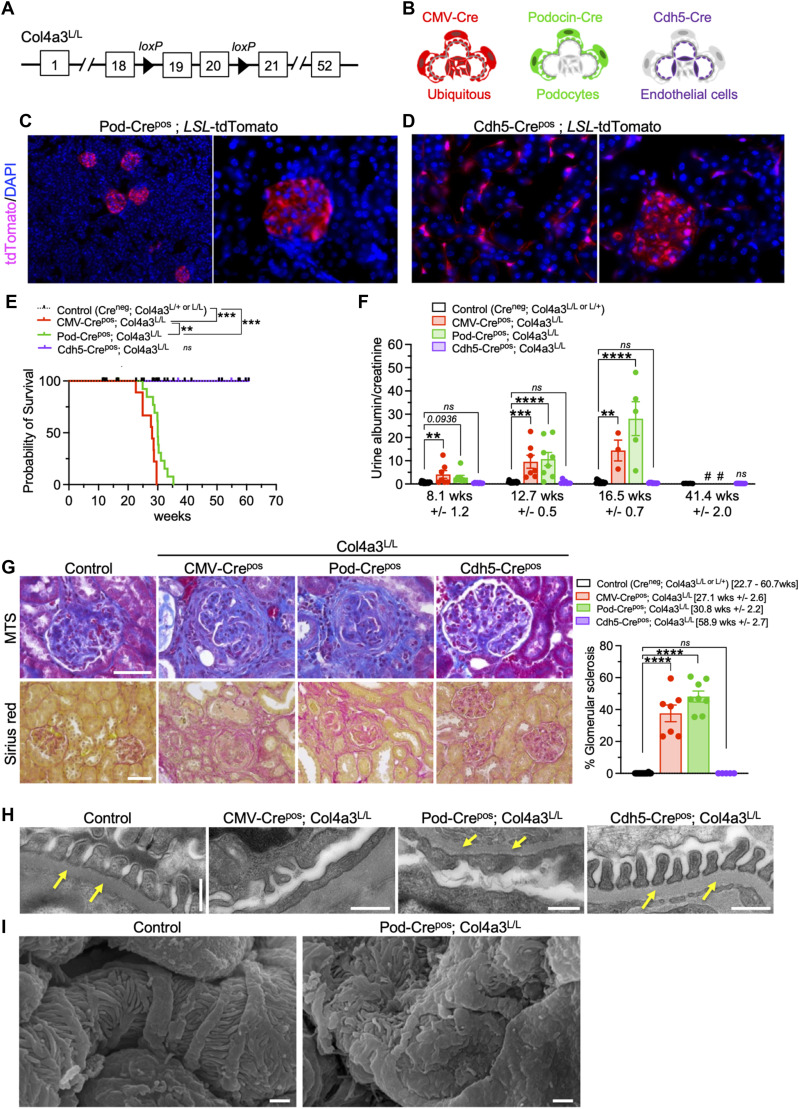
Conditional deletion of Col4a3 in podocytes results in renal failure. **(A)** Schematic representation of the Col4a3 conditional deletion allele with Cre recombinase–mediated excision of exons 19 and 20. **(B)** Schematic representation of cells expressing the listed Cre recombinase transgenes. **(C)** Representative images of the tdTomato expression reporter in kidney sections from Pod-Cre^pos^; *LSL*-tdTomato. Scale bars: left, 50 μm; right 20 μm. **(D)** Representative images of the tdTomato expression reporter in kidney sections from Cdh5-Cre^pos^; *LSL*-tdTomato. Scale bar: 20 μm. **(E)** Survival of mice in the indicated genotypes. Log-rank (Mantel–Cox) test. Control (Cre^neg^; Col4a3^L/+ or L/L^), n = 47; CMV-Cre^pos^; Col4a3L^/L^, n = 9; Pod-Cre^pos^; Col4a3^L/L^, n = 23; Cdh5-Cre^pos^; Col4a3^L/L^, n = 17 mice. **(F)** Proteinuria expressed as urine albumin over creatinine ratio in the indicated genotypes over time. One-way ANOVA with Dunnett’s multiple-comparisons test at each time point. At 8.1 wk (±1.2 wk): control (Cre^neg^; Col4a3^L/+ or L/L^), n = 15; CMV-Cre^pos^; Col4a3L^/L^, n = 7; Pod-Cre^pos^; Col4a3^L/L^, n = 8; Cdh5-Cre^pos^; Col4a3^L/L^, n = 8 mice. At 12.7 wk (±0.5 wk): control (Cre^neg^; Col4a3^L/+ or L/L^), n = 18; CMV-Cre^pos^; Col4a3L^/L^, n = 7; Pod-Cre^pos^; Col4a3^L/L^, n = 8; Cdh5-Cre^pos^; Col4a3^L/L^, n = 7 mice. At 16.5 wk (±0.7 wk): control (Cre^neg^; Col4a3^L/+ or L/L^), n = 14; CMV-Cre^pos^; Col4a3L^/L^, n = 3; Pod-Cre^pos^; Col4a3^L/L^, n = 5; Cdh5-Cre^pos^; Col4a3^L/L^, n = 10 mice. At 41.4 wk (±2.0 wk): control (Cre^neg^; Col4a3^L/+ or L/L^), n = 5; Cdh5-Cre^pos^; Col4a3^L/L^, n = 10 mice. #, no mice alive at that age. **(G)** Representative MTS and sirius red (SR)–stained glomeruli of control (MTS, 28.7 wk; SR, 29.7 wk), CMV-Cre^pos^; Col4a3L^/L^ (MTS, 28.1 wk; SR, 29.6 wk), Pod-Cre^pos^; Col4a3^L/L^ (MTS and SR, 33.3 wk), and Cdh5-Cre^pos^; Col4a3^L/L^, (MTS and SR, 60.7 wk). Scale bar: 50 μm. Bar graphs depicts percent glomerular sclerosis in the indicated groups and age brackets. One-way ANOVA with Holm–Sidak’s multiple-comparisons test. Control (Cre^neg^; Col4a3^L/+ or L/L^), n = 22; CMV-Cre^pos^; Col4a3L^/L^, n = 7; Pod-Cre^pos^; Col4a3^L/L^, n = 8; Cdh5-Cre^pos^; Col4a3^L/L^, n = 5 mice. **(H)** Representative transmission electron microscopy images of the indicated genotypes. Control (Cre^neg^; Col4a3^L/+^), 29.7 wk; CMV-Cre^pos^; Col4a3L^/L^, 29.6 wk; Pod-Cre^pos^; Col4a3^L/L^, 28.4 wk; Cdh5-Cre^pos^; Col4a3^L/L^, 36.9 wk. Scale bar: 0.5 μm. Yellow arrows point to the GBM. **(I)** Representative scanning electron microscopy images of the indicated genotypes at 16.4 wk, control: Pod-Cre^neg^; Col4a3^L/L^. Scale bar: 1 μm. **P < 0.01, ***P < 0.001, ****P < 0.0001, *ns*: not significant or otherwise listed. Source data are available for this figure.

**Figure S1.**
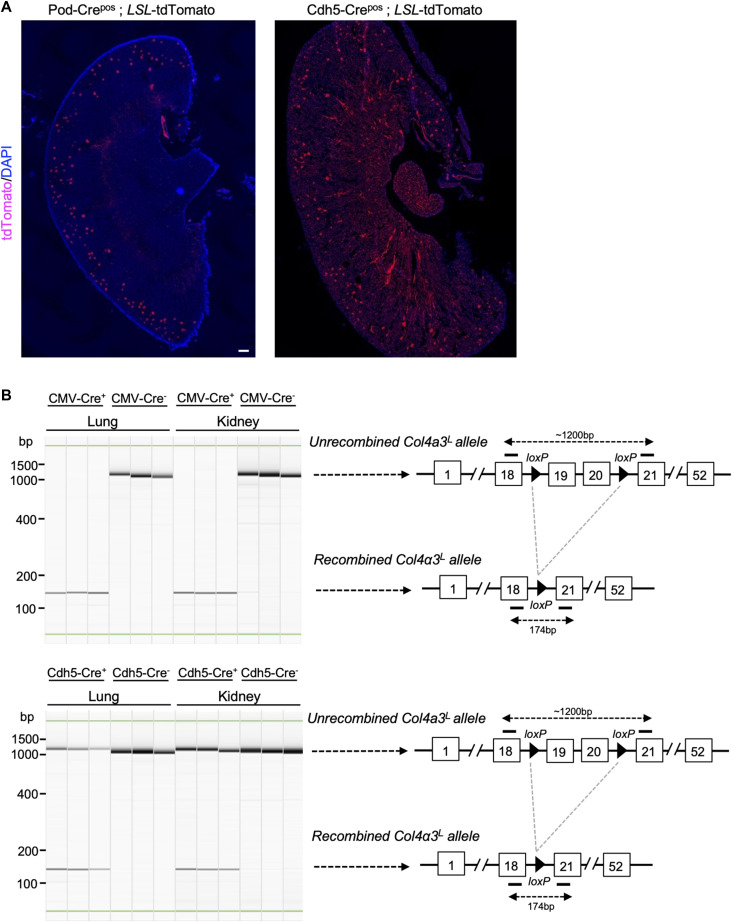
Validation of Cre reporters and Col4a3^L/L^ alleles. **(A)** Representative images of the tdTomato expression reporter in kidney sections from the indicated genotypes. Scale bars: left, 300 μm. **(B)** Representative images of electrophoretic migration of PCR products from the indicated schematic representation in the listed tissues and genotypes.

**Figure S2.**
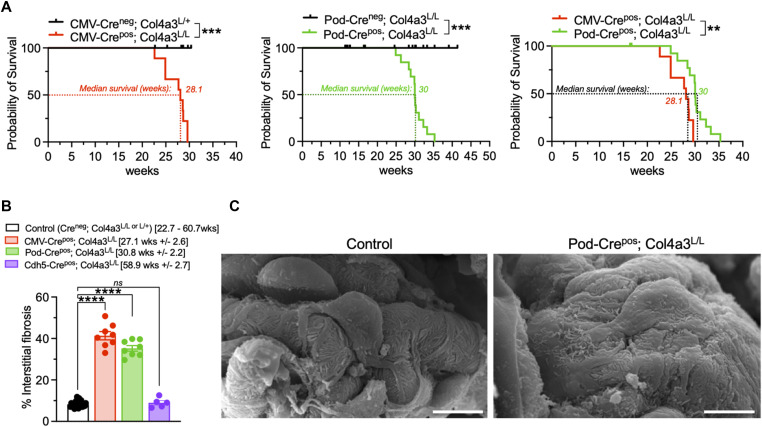
Characterization of the Pod-Crepos; Col4a3L/L phenotype. **(A)** Survival of mice in the indicated genotypes further subdivided from Fig 1E. Log-rank (Mantel–Cox) test. CMV-Cre^neg^; Col4a3^L/+ or L/L^, n = 8; CMV-Cre^pos^; Col4a3L^/L^, n = 9; Pod-Cre^neg^; Col4a3^L/L^, n = 26; Pod-Cre^pos^; Col4a3^L/L^, n = 23 mice. **(B)** Percent interstitial fibrosis in the indicated groups and age brackets. One-way ANOVA with Holm–Sidak’s multiple comparison test. Control (Cre^neg^; Col4a3^L/+ or L/L^), n = 23; CMV-Cre^pos^; Col4a3L^/L^, n = 8; Pod-Cre^pos^; Col4a3^L/L^, n = 8; Cdh5-Cre^pos^; Col4a3^L/L^, n = 5 mice. **(C)** Representative scanning electron microscopy images in the indicated genotypes at 16.4 wk. Scale bar: 5 μm. **P < 0.01, ***P < 0.001, ****P < 0.0001, *ns*, not significant. Source data are available for this figure.

The GBM is juxtaposed by podocytes and endothelial cells, with both cell types presumed to contribute to type IV collagen production and its turnover. While systemic loss of *Col4a3* in Col4a3^KO^ mice recapitulates the GBM defects associated with Alport syndrome, the precise cellular contribution of type IV collagen chains in the GBM remains unknown. We generated a novel conditional allele for the deletion of *Col4a3* (Fig 1A), which enabled the ubiquitous *Col4a3* deletion (CMV-Cre), podocyte-specific deletion (Podocin-Cre; Pod-Cre), or endothelial specific deletion (Cdh5-Cre) of *Col4a3* (Fig 1B). Lineage tracing studies with the tdTomato reporter allele confirmed the fidelity of the Pod-Cre and Cdh5-Cre alleles (Figs 1C and D and S1A), consistent with previous reports (Kanasaki et al, 2008; Lovisa et al, 2020). Recombination PCR analyses confirmed the Cre-mediated recombination of the engineered *Col4a3* allele (Fig S1B). Systemic deletion of *Col4a3* (CMV-Crepos; Col4a3^L/L^) phenocopied the total body knock out of *Col4a3* (Col4a3^KO^) (Cosgrove et al, 2007; LeBleu et al, 2009), demonstrating a median survival of 28.1 wk and death due to renal failure (Figs 1E and S2A). Renal function impairment was evidenced with progressive proteinuria and histopathological findings, including glomerular sclerosis and tubular interstitial fibrosis (Figs 1F and G and S2B).

Specific deletion of *Col4a3* in podocytes (Pod-Crepos; Col4a3^L/L^) presented with a phenotype similar to the ubiquitous deletion of *Col4a3* (CMV-Crepos; Col4a3^L/L^), with a median survival of 30 wk (Figs 1D and S2A), significant proteinuria (Fig 1E), and histopathological findings associated with glomerular disease (Figs 1G and S2B). In contrast, deletion of *Col4a3* in endothelial cells (Cdh5-Crepos; Col4a3^L/L^) did not result in glomerular disease, with no impact on survival, renal function, and kidney histopathology (Figs 1E–G and S2A and B). Transmission electron microscopy analyses showed splitting, thinning, and thickening of the GBM in both CMV-Crepos; Col4a3^L/L^ and Pod-Crepos; Col4a3^L/L^ kidneys (Fig 1H). Podocyte foot process effacement was noted on scanning electron microscopy analyses of Pod-Crepos; Col4a3^L/L^ kidneys (Figs 1I and S2C). These findings are in contrast with the intact GBM observed in control and Cdh5-Crepos; Col4a3^L/L^ kidneys (Fig 1H). Taken together, these results support the notion that podocytes are rate-limiting producers of the type IV collagen chains responsible for the type IV collagen composition in the developing and mature GBM.

### Horizontal gene transfer to podocyte following bone marrow transplantation (BMT) rescues Col4a3 production in Col4a3^KO^ mice

**Figure 2.**
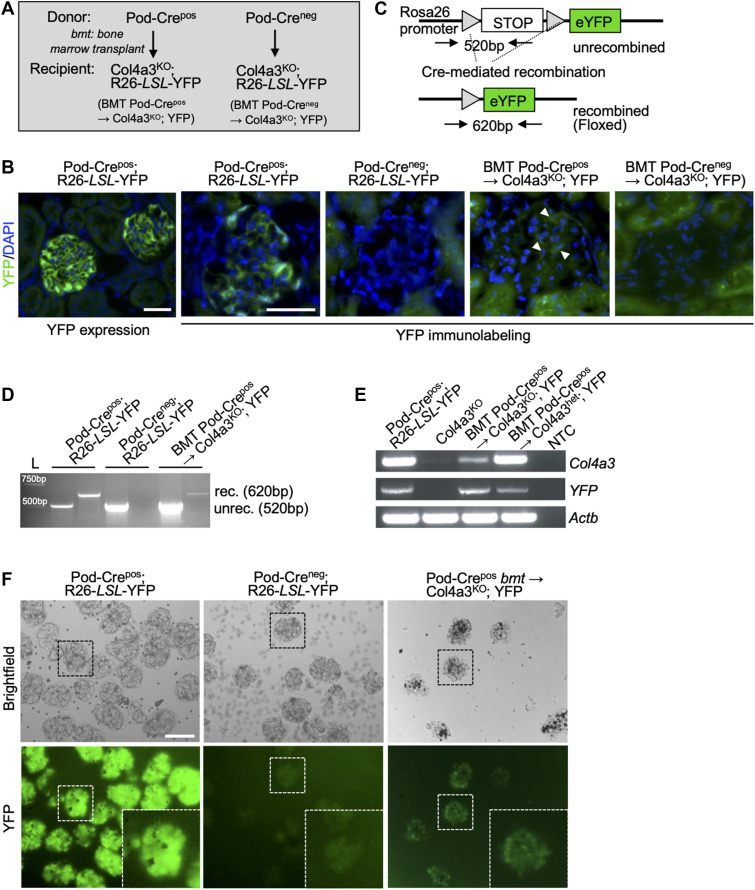
Evidence of bone marrow–derived cell fusion with podocytes in Col4a3^KO^ mice. **(A)** Schematic of the bone marrow transplant strategy. **(B)** Representative images of YFP endogenous expression or YFP immunolabeling with DAPI nuclear stain of the kidney sections from the indicated groups. Arrowheads point to YFP positive cells. Scale bar: 50 μm. **(C)** Schematic representation of the YFP allele recombination and PCR strategy. **(D)** Electrophoretic migration of PCR products amplified from kidney DNA taken from the indicated groups. rec: recombined (floxed) YFP allele, unrec: unrecombined YFP allele. **(E)** Electrophoretic migration of PCR products from kidney cDNA prepared from the indicated groups showing expression of *Col4a3*, *YFP*, and internal control *Actb*. **(F)** Representative bright-field and endogenous YFP images of purified glomeruli from the indicated group with digital zoom inset. Scale bar: 100 μm.

**Figure S3.**
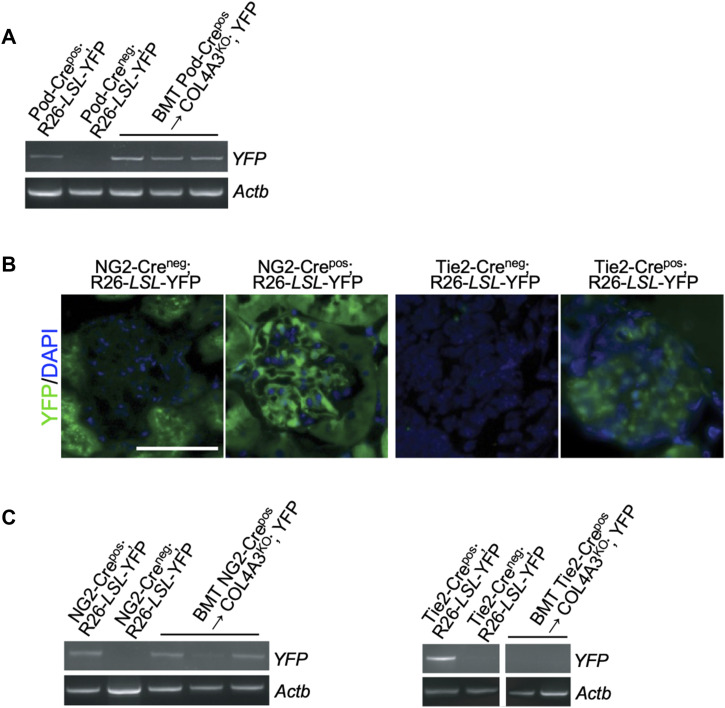
Glomerular cell horizontal gene transfer with reporter alleles. **(A)** Electrophoretic migration of PCR product from kidneys cDNA of the indicated group showing expression of *YFP* and internal control *Actb*. **(B)** Representative endogenous YFP images of glomeruli from the indicated groups. Scale bar: 50 μm. **(C)** Electrophoretic migration of PCR products amplified from kidney cDNA prepared from the indicated groups showing expression of *YFP* and internal control *Actb*.

Previous studies reported on de novo *Col4a3* expression in the GBM of Col4a3^KO^ mice following transplantation with Col4a3WT donor bone marrow (Prodromidi et al, 2006; Sugimoto et al, 2006; LeBleu et al, 2009). The improved renal function, histopathology, and survival correlated with restoration of *Col4a3* expression. Col4a3^KO^ bone marrow transplantation in Col4a3^KO^ mice did not impact the renal disease (Prodromidi et al, 2006; Sugimoto et al, 2006; LeBleu et al, 2009). To define the fate of the stem cells homing to the injured glomeruli with concomitant expression of missing *Col4a3* to rescue the GBM defect, we engineered mice to explore bone marrow donor cell differentiation into podocytes and/or fusion (or horizontal gene transfer) with recipient damaged podocytes. We generated Col4a3^KO^ mice harboring the reporter allele R26-LSL-eYFP (Col4a3^KO^; YFP) and transplanted them with bone marrow from Col4a3^WT^ donors with and without expression of the Pod-Cre transgene (Pod-Cre^pos^ and Pod-Cre^neg^, Fig 2A). Bone marrow–derived cell horizontal gene transfer in Col4a3^KO^; YFP mice transplanted with Pod-Cre^pos^ bone marrow (Pod-Cre^pos^ *bmt*→ Col4a3^KO^; YFP) was observed in the glomeruli of kidney sections immunolabeled for YFP (Fig 2B). A PCR strategy for the amplification of the recombined R26-LSL-eYFP allele also indicated that horizontal gene transfer, possibly including cell fusion, had occurred following BMT (Fig 2C). PCR amplification for the Cre-mediated recombined R26-LSL-YFP allele was observed in positive control (Pod-Cre^pos^; YFP) and Pod-Cre^pos^ *bmt* → Col4a3^KO^; YFP kidneys (Fig 2D). In contrast, Pod-Cre^neg^; YFP mice did not show R26-LSL-YFP allele recombination by PCR, supporting the fidelity and specificity of this in vivo cell fate-mapping strategy (Fig 2D). *Col4a3* transcripts were detected following Col4a3^WT^ bone marrow transplant into Col4a3^KO^ mice (Fig 2E). YFP transcripts were also detected (Figs 2F and S3A), providing further evidence of horizontal gene transfer. Increased YFP expression levels were noted when Col4a3^KO^ mice were transplanted with Col4a3^WT^ bone marrow compared with healthy Col4a3 heterozygous (Col4a3^het^) recipient mice that were similarly transplanted (Fig 2E). This finding suggests that healthy glomeruli can transfer genetic material or fuse with bone marrow–derived cells in the timeframe of our experiments. As the expression of YFP is under the constitutive ROSA promoter, this result also indicates that diseased glomeruli possibly yield more horizontal gene transfer or cell fusion events than healthy ones. Increased horizontal gene transfer or cell fusion in diseased glomeruli may reflect more efficient recruitment of bone marrow–derived cells, or possibly that the resulting heterokaryon in the case of cell fusion is long lived. Purified glomeruli from positive control (Pod-Cre^pos^; YFP) and Pod-Cre^pos^ *bmt* → Col4a3^KO^; YFP mice showed endogenous YFP expression in glomeruli, indicating horizontal gene transfer or cell fusion with injured podocytes following bone marrow transplantation (Fig 2F).

To ascertain whether horizontal gene transfer occurred in other glomerular cells beyond podocytes, Col4a3^KO^; YFP mice were transplanted with bone marrow from mesangial (NG2-Cre) and endothelial/macrophage (Tie2-Cre) specific transgenic mice. Endogenous glomerular YFP expression reflective of the respective transgenes was confirmed in kidney sections from both NG2-Cre^pos^; YFP and Tie2-Cre^pos^; YFP mice compared with respective Cre^neg^ controls (Fig S3B). YFP transcripts were detected in the kidney of Col4a3^KO^; YFP mice transplanted with NG2-Cre^pos^ transgene, suggesting that mesangial cells and/or perivascular cells (that would also be captured with this transgene) may also receive genetic material from bone marrow–derived cells (Fig S3C). In contrast, YFP transcripts were not detected in endothelial cells and monocytes traced using the Tie2-Cre^pos^ transgene during the timeframe of this experiment (Fig S3C).

**Figure S4.**
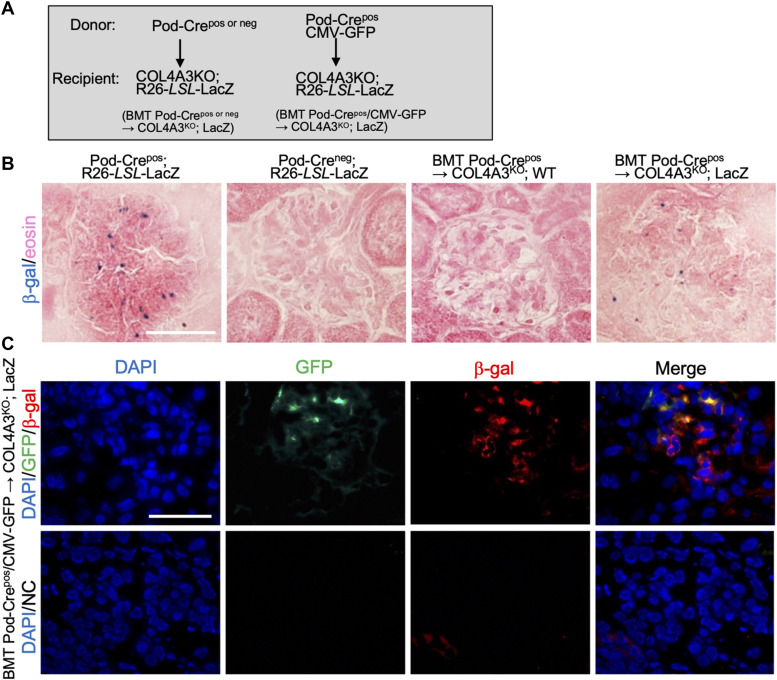
Capture of fusion with bone marrow transplant in Pod-Crepos; Col4a3L/L mice. **(A)** Schematic of the bone marrow transplant strategy. **(B)** Representative images of LacZ staining of the kidney sections from the indicated groups. Scale bar: 50 μm. **(C)** Representative images of GFP and ß-galactosidase staining with DAPI nuclear stain of the kidney sections from the indicated groups. Scale bar: 50 μm.

Podocyte horizontal gene transfer or cell fusion with bone marrow–derived cells was confirmed with an additional strategy that used the R26-LSL-LacZ (LacZ) reporter allele (Fig S4A). ß-galactosidase (ß-gal) was detected in the glomeruli of Col4a3^KO^; LacZ–recipient mice transplanted with Pod-Cre^pos^ bone marrow, which was not the case in Col4a3^KO^ recipients (lacking the LacZ allele) that were transplanted with Pod-Cre^pos^ bone marrow (Fig S4B). To ascertain the relative contribution of horizontal gene transfer or cell fusion and cell differentiation in the repair of GBM in the bone marrow transplanted Col4a3^KO^; LacZ mice, donor bone marrow from dual transgenic mice expressing both Pod-Cre and CMV-GFP were used. GFP and ß-gal immunolabeling of the kidney glomeruli showed minimal GFP single positive cells (capturing differentiation) in comparison with GFP and β-gal double positive cells (capturing fusion, Fig S4C). Taken together, these results illustrate that the therapeutic bone marrow transplant in Col4a3^KO^ mice is realized, at least in part, via the horizontal gene transfer or cell fusion of bone marrow cells with recipient injured podocytes. The results also suggest that the transcriptome of donor bone marrow cells is modified to gain expression of the transgene (Pod-Cre), possibly after the formation of the heterokaryon in podocytes.

### BM MSCs infusion rescues Col4a3^KO^ mice and transfer genetic material to podocytes in a TGFß1-dependent manner

**Figure 3.**
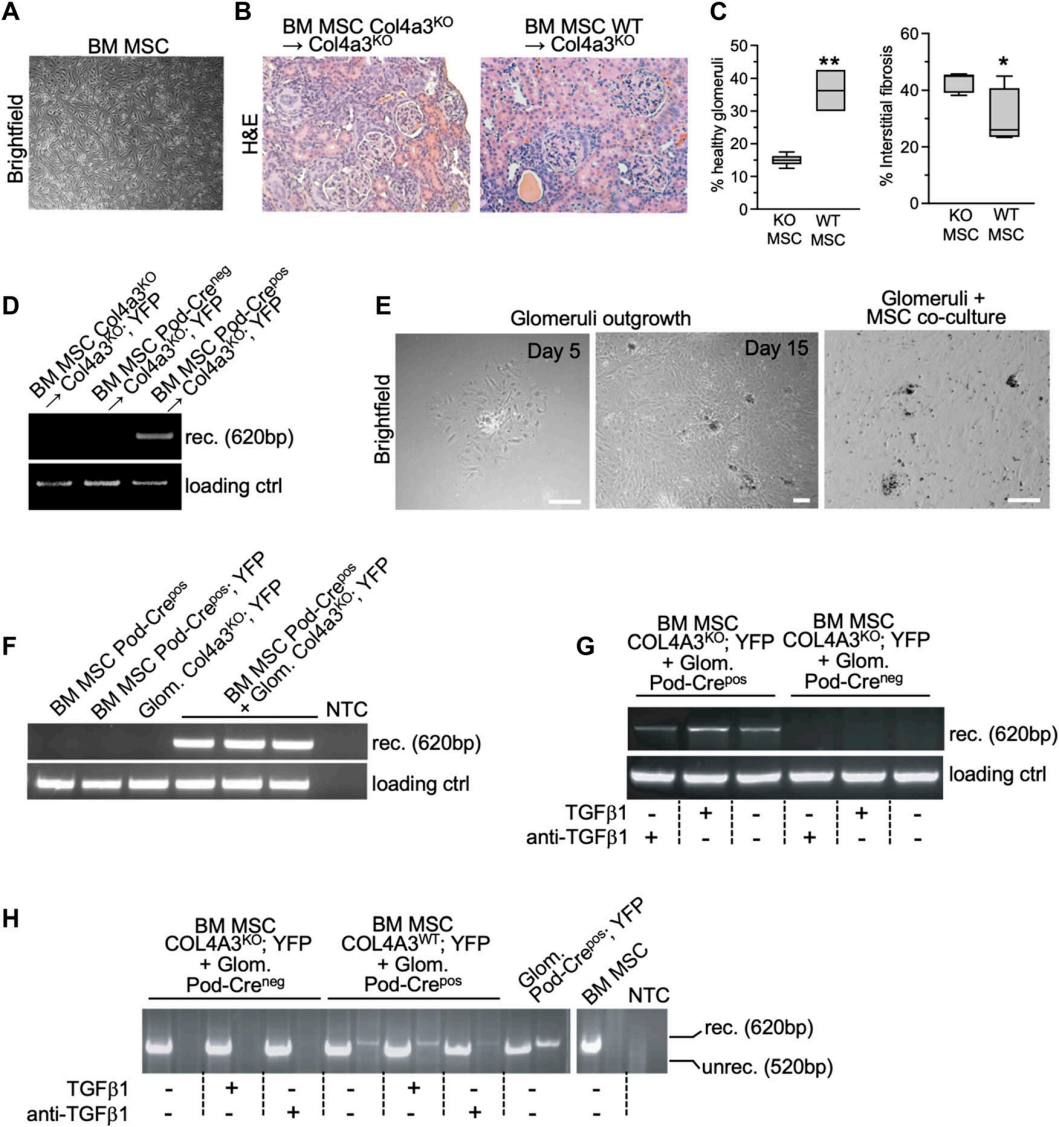
Bone marrow–derived stem cells fuse with podocyte to rescue renal damage in Col4a3KO mice. **(A)** Representative bright-field image of murine bone marrow–derived stem cells. Scale bar: 200 μm. **(B)** Representative H&E images of the kidneys of mice in the listed groups. Scale bar: 100 μm. **(C)** Histopathological assessments (percent healthy glomeruli and interstitial fibrosis) of the kidneys of mice in the listed groups. Col4a3^KO^; YFP administered with Pod-Cre^pos^ MSC (WT MSC), n = 4; control Col4a3^KO^; YFP administered with Pod-Cre^neg^ MSC (WT MSC), n = 5. Unpaired *t* test. **(D)** Electrophoretic migration of PCR products amplified from kidney DNA prepared from the indicated groups. rec: recombined (floxed) YFP allele. Loading ctrl: loading control. **(E)** Representative bright-field images of glomeruli in culture for 5 and 15 d, and in co-culture with bone marrow–derived MSC. Scale bar: 100 μm. **(F, G, H)** Electrophoretic migration of recombination PCR products amplified from DNA prepared from glomeruli of the indicated groups (+/− MSC co-culture). rec: recombined (floxed) YFP allele. Loading ctrl: loading control. *P < 0.05, **P < 0.01. Source data are available for this figure.

**Figure S5.**
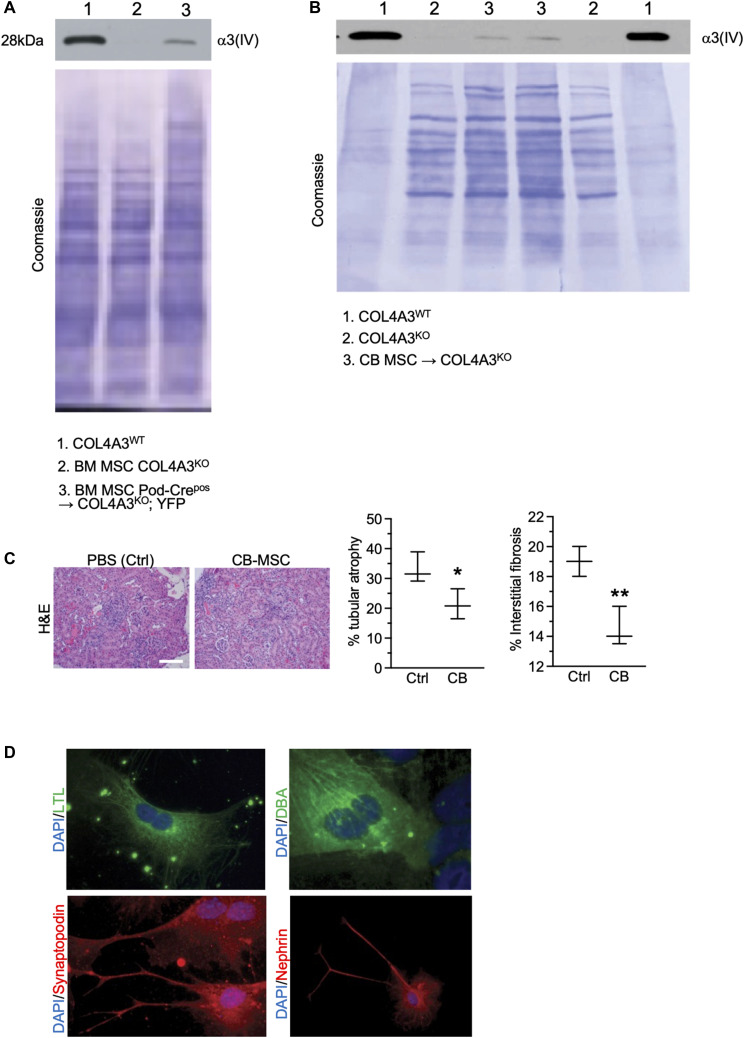
Expression of Col4a3 in treated Col4a3KO mice and utility of cord blood-derived MSC (CB-MSC). **(A, B)** Western blot for Col4a3 in kidney ECM from the indicated groups and Coomassie-stained membrane showing loading of proteins in each lane. **(C)** Representative images of H&E–stained kidneys in the indicated groups and associated histopathological scoring. n = 3 mice per group. Scale bar: 50 μm. **(D)** Representative images of immunolabeled iPSC undergoing differentiation. Nuclei are stained with DAPI. Source data are available for this figure.

Informed by the bone marrow transplant studies detailed above, we next queried which BM-derived cells demonstrate horizontal gene transfer or cell fusion with podocytes and rescue the Alport phenotype in Col4a3^KO^ mice. Our previous studies indicated that unfractionated wild-type bone marrow infusion rescued the renal phenotype in Col4a3^KO^ mice compared with unfractionated Col4a3^KO^ bone marrow cell infusion (LeBleu et al, 2009). We also reported on BMT studies in Col4a3^KO^ mice, in which transplantation with CD11b^KO^ and Rag1^KO^ donor bone marrow failed to provide evidence that a hematological lineage in the bone marrow–derived cells is responsible for the phenotypic rescue in Col4a3^KO^ mice (LeBleu et al, 2009). We next evaluated whether a mesenchymal bone marrow lineage is functionally implicated in Col4a3^KO^ mice rescue. Bone marrow–derived MSC were allowed to adhere and expand in vitro (Fig 3A). Col4a3^KO^ mice (7–13 wk old) were treated with a single intravenous infusion of MSC from Col4a3^WT^ (WT) or Col4a3^KO^ donor mice (controls). Histopathological findings (19–23 wk old) indicated a significant improvement in glomerular health and suppression of interstitial fibrosis in Col4a3^WT^ MSC-treated mice compared with Col4a3^KO^ MSC-treated mice (Fig 3B and C). The phenotypic rescue was associated with de novo detection of *Col4a3* protein in the kidneys in Col4a3^KO^ mice treated with Col4a3^WT^ MSC (Fig S5A). We also purified and tested human cord blood–derived MSC (CB-MSC). Human MSC from BM and adipocytes survive in immunocompetent mice if kept undifferentiated (Niemeyer et al, 2008). A single systemic injection of CB-MSC resulted in de novo expression of *Col4a3* in Col4a3^KO^ mice and an improvement in glomerular histopathology, albeit in a very limited number of recipient mice (Fig S5B and C).

The MSC infusion experiments were also designed to interrogate their horizontal gene transfer or cell fusion potential with podocytes. MSC from the bone marrow of Pod-Cre^pos^, Pod-Cre^neg^, and Col4a3^KO^ mice were systemically administered into Col4a3^KO^; YFP recipient mice. Recombination of the YFP allele in the kidneys of Col4a3^KO^; YFP mice was observed following infusion of the mice with Pod-Cre^pos^ MSC, in contrast to the case with control Col4a3^KO^; YFP mice that were treated with MSC from either Col4a3^KO^ or Pod-Cre^neg^ mice (Fig 3D). The horizontal gene transfer or cell fusion of MSC was studied in vitro using co-culture of MSC with purified glomeruli (Fig 3E). In co-culture, YFP allele recombination, indicative of horizontal gene transfer or cell fusion, was detected when MSC harbored the Pod-Cre^pos^ transgene, in contrast to the case when MSC and glomeruli were cultured independently from one another (Fig 3F). MSCs from Pod-Cre^pos^; YFP bone marrow did not demonstrate YFP recombination (Fig 3F), indicating that the expression of the Pod-Cre transgene occurs following horizontal gene transfer or cell fusion with glomeruli rather than from possible differentiation into podocytes in vitro.

Given the enhanced YFP expression in bone marrow transplanted Col4a3^KO^ versus Col4a3^Het^ mice, possibly representing a greater number of horizontal gene transfer or cell fusion events (Fig 2E), we posited that the response to injury in Col4a3^KO^ kidneys may represent a glomerular microenvironment permissive for horizontal gene transfer or cell fusion. Renal TGFß1 is up-regulated in fibrosis and plays both a profibrotic and protective function (Sureshbabu et al, 2016). TGFß1 acts as a molecular switch in podocytes, regulating differentiation, proliferation, or survival in a context-dependent manner (Wu et al, 2005; Sureshbabu et al, 2016; Frank et al, 2022). We observed increased levels of YFP recombination (indicative of an increased number of horizontal gene transfer or cell fusion events) when Col4a3^KO^; YFP MSC and Pod-Cre^pos^ glomeruli were co-cultured with TGFß1, and a reduced level of YFP recombination when the MSC/glomeruli cultures were treated with TGFß1 neutralizing antibodies (anti-TGFß1) (Fig 3G). In this setting, the use of Pod-Cre^pos^ glomeruli rather than Col4a3^KO^; YFP glomeruli minimized the potential increase in TGFß1 production in vitro from Col4a3^KO^ damaged glomeruli compared with Col4a3^WT^ (Pod-Cre^pos^) glomeruli ex vivo. The results also showed that recombination events were observed with Cre-mediated recombination of the reporter YFP allele in glomeruli following co-culture with Pod-Cre^pos^ MSCs (Fig 3F) and with Cre-mediated recombination of the reporter YFP allele in MSCs following co-culture with Pod-Cre^pos^ glomeruli (Fig 3G and H). Taken together, these results suggest that TGFß1 likely promotes horizontal gene transfer or cell fusion of MSC with glomerular podocytes and that the recipient cell subsequently undergo Cre-mediated recombination of its DNA independent of the origin (donor or host cells) of the transgene and reporter allele, and independent of the Col4a3 genetic status.

### iPSC infusion rescues Col4a3^KO^ mice and shows horizontal gene transfer with podocytes

**Figure 4.**
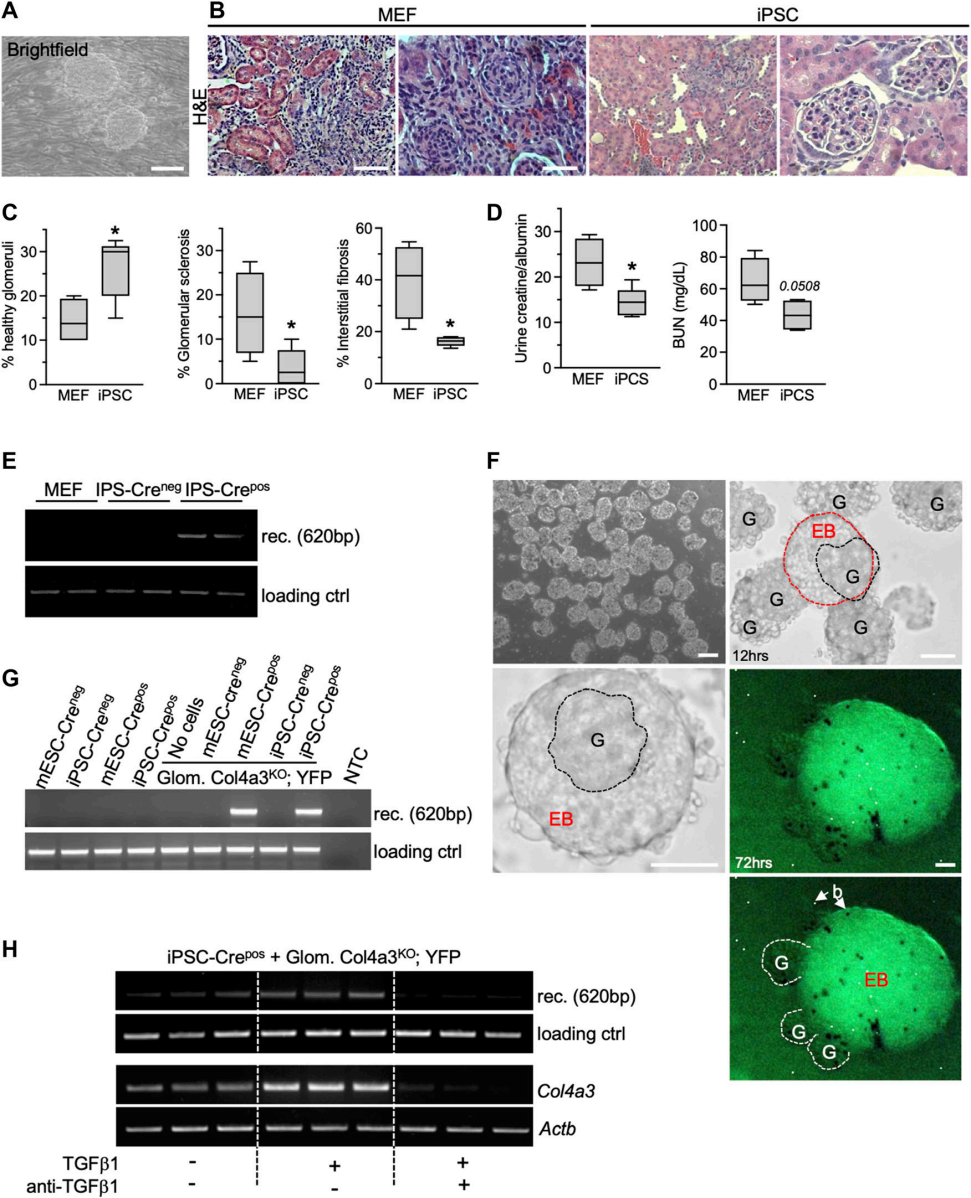
iPSC fuse with podocyte and rescue renal damage in Col4a3KO mice. **(A)** Representative bright-field image of murine iPSC on MEF feeder layer. Scale bar: 100 μm. **(B)** Representative H&E images of the kidneys of mice in the listed groups. Left panel, scale bar: 100 μm; right panel, scale bar: 50 μm. **(C)** Histopathological assessments (percent healthy glomeruli, glomerular sclerosis, and interstitial fibrosis) of the kidneys of mice in the listed groups. MEF treated, n = 4; iPSC, n = 5. Unpaired *t* test. **(D)** Renal function evaluated by proteinuria (urine creatinine/albumin ratio) and blood urea nitrogen levels in the indicated groups. Unpaired *t* test. **(E)** Electrophoretic migration of PCR products amplified from kidney DNA extracted from the indicated groups. rec: recombined (floxed) YFP allele. Loading ctrl: loading control. **(F)** Representative bright-field images of purified glomeruli, and glomeruli in co-culture with iPSC for 12 and 72 h. G, glomerulus; EB, embryonic body; B, beads. Left upper panel, scale bar: 100 μm; other scale bars: 50 μm. **(G)** Electrophoretic migration of recombination PCR products amplified from DNA extracted from glomeruli co-cultured with iPSC or mESC. rec: recombined (floxed) YFP allele. Loading ctrl: loading control. **(H)** Electrophoretic migration of recombination PCR products amplified from DNA extracted from glomeruli co-cultured with iPSC (top). rec: recombined (floxed) YFP allele. Loading ctrl: loading control; and electrophoretic migration of PCR products amplified from cDNA prepared from glomeruli co-cultured with iPSC in the presence or absence TGFβ1 or anti-TGFβ1 antibodies as indicated, showing relative expression of *Col4a3* and internal control *Actb* (bottom). rec: recombined (floxed) YFP allele. Loading ctrl: loading control. *P < 0.05 unless otherwise indicated. Source data are available for this figure.

Next, we ascertained whether iPSC offered a similar therapeutic benefit reported above with MSC via horizontal gene transfer or cell fusion with podocytes. Although iPSC were readily able to differentiate in vitro into renal lineages including synaptopodin- and nephrin-expressing cells (Fig S5D and as previously described [Song et al, 2012]), we tested the therapeutic potential of undifferentiated iPSC. A single-cell suspension of undifferentiated iPSC was administered systemically into Col4a3^KO^ mice. In contrast with the MEF feeder layer, iPSC infusion induced kidney regeneration with improved kidney function and glomerular histopathology (Fig 4A–D). We engineered iPSC to express Cre recombinase (EF1α-EGFPCre) to test their ability to transfer genetic material or fuse with podocytes. iPSC that expressed Cre recombinase (iPSC-Cre^pos^), control IPS (iPSC-Cre^neg^), and MEF control were infused systemically into Col4a3^KO^; YFP mice. The horizontal gene transfer or cell fusion of iPSC with podocytes was demonstrated with detection of the recombined YFP allele specifically in the kidneys of Col4a3^KO^; YFP mice treated with iPSC-Crepos (Fig 4E). No detection was observed in mice treated with MEF or iPSC-Creneg cells (Fig 4E). Evidence of horizontal gene transfer or cell fusion was tested in vitro using glomeruli from Col4a3^KO^; YFP mice that were co-cultured with undifferentiated iPSC and mouse embryonic stem cells (mESC), with or without Cre recombinase expression, respectively. iPSC and mESC rapidly aggregated into embryoid bodies (EB), seen as early as 12 h following co-culture with purified kidney glomeruli (Fig 4F). The EBs partly engulfed the glomeruli and grew into larger EBs after 72 h of co-culture (Fig 4F). PCR analyses from the DNA of the cells in these co-cultures showed recombination of the YFP allele when iPSC or mESC expressed Cre recombinase (Fig 4G), indicative of horizontal gene transfer or cell fusion. Horizontal gene transfer was enhanced in iPSC-Cre^pos^ and Col4a3^KO^; YFP glomeruli co-cultured in the presence of TGFß1, and such events were reduced in the presence of anti-TGFß1 antibodies (Fig 4H). The relative expression of *Col4a3* was increased in iPSC-Cre^pos^ and Col4a3^KO^; YFP glomeruli co-cultured with TGFß1 and was reduced upon addition of anti-TGFß1 antibodies, suggesting that an increased number of horizontal gene transfer or cell fusion events is associated with increased expression of the missing Col4a3 chain (Fig 4H).

## Discussion

We previously reported on several cell-based approaches to restore the type IV collagen network necessary for GBM function in murine models of Alport syndrome (Col4a3^KO^ mice) (Sugimoto et al, 2006; LeBleu et al, 2009). Here we have specifically investigated bone marrow–derived MSC and iPSC in the treatment of mice with the genetic defect associated with Alport syndrome. We also report on a novel genetically engineered allele to reveal that podocytes are the dominant producers of type IV collagen in the developing kidney GBM. Our results also show that loss of *Col4a3* in endothelial cells is insufficient to generate the GBM defect associated with Alport syndrome, underscoring the critical role for podocytes in GBM type IV collagen production. These findings are in agreement with reports that used transgenes to rescue the type IV collagen network in Col4a3^KO^ mice, which showed that expression of the missing type IV collagen in podocytes (Lin et al, 2014), but not in endothelial cells (Funk et al, 2019), is sufficient to restore GBM function. Our genetic approach shows that podocyte horizontal gene transfer or cell fusion with exogenously administered unfractionated bone marrow cells, bone marrow–derived MSC or iPSC was associated with in de novo expression of the missing type IV collagen and improves renal outcome in Col4a3^KO^ recipient mice. Whereas the use of undifferentiated iPSC is unlikely to have clinical utility, our proof-of-concept experiments support an undifferentiated state of donor cells may be a feature necessary for successful horizontal gene transfer or cell fusion. Further study and precise definition of cell sources and state would be necessary to advance cell-based therapy for Alport syndrome.

Horizontal transfer of genetic material in vivo was previously reported in distinct pathologies using extracellular vesicles or nanotubes (Burghoff et al, 2008; Tome et al, 2009; Uygur et al, 2019; Barutta et al, 2021). Beyond horizontal transfer of a limited amount of genetic material, cell fusion with the formation of a sable heterokaryon capable of genetic rescue remains challenging to ascertain. Prior studies also inform on the potential of bone marrow–derived cells generating possible heterokaryon with podocytes (Poulsom et al, 2003; Prodromidi et al, 2006). Cell fusion in adult tissue is reported as a rare biological event in the liver (Alvarez-Dolado et al, 2003; Vassilopoulos et al, 2003; Fujimiya et al, 2007), neuronal tissues (Johansson et al, 2008), and skeletal muscle regeneration (Andrade et al, 2005). Bone marrow–derived cells appear to adopt the phenotype of the cells they fuse with (Terada et al, 2002) and spontaneous cell fusion appears to promote genetic reprogramming (Ying et al, 2002; Ambrosi & Rasmussen, 2005; Alvarez-Dolado, 2007; Bonde et al, 2010). Our study indicates that the expression of the podocyte lineage transgene (Podocin-Cre) was induced in MSC following fusion with podocytes and that horizontal gene transfer or cell fusion events, as evidenced by PCR-based capture of recombined reporter alleles, were increased by TGFβ1. Inflammatory processes are known to promote cell fusion (Johansson et al, 2008; Singec & Snyder, 2008; Davies et al, 2009), and our studies suggest that TGFβ1—an inflammatory Th2 cytokine—likely mediates cell fusion in this setting by bringing donor and recipient cells into a close proximity, or by serving as a direct catalyst of heterotypic cell fusion. Irradiation of tissue, promoting inflammatory responses, may also partake in enhancing cell fusion. iPSC also demonstrated horizontal gene transfer/cell fusion with glomerular epithelial cells in a TGFβ1-dependent manner to induce genetic reprogramming and facilitate kidney regeneration. The host-directed expression of the donor *Col4a3* gene following fusion of stem cells was associated with a dynamic gene expression reprogramming controlled in part by TGFβ1. Notably, TGFβ1 has been implicated in the differentiation of podocytes and cell cycle progression (Wu et al, 2005; Frank et al, 2022), which may be more permissive to reprogramming of gene expression following cell fusion. Collectively our studies inform on the association of horizontal gene transfer or cell fusion with the rescue of the renal phenotype; however, it remains unknown from our studies how much of the horizontal gene transfer/cell fusion is directly responsible for de novo Col4a3 expression.

Our experiments suggest that the genetic information of the “donor” stem cell was modified by the “recipient host” cell to facilitate a combined genetic program. The significant kidney regenerative potential of iPSC in this setting is likely realized because of the generation of heterokaryons which allow for a corrective gene expression reprogramming, restoring *Col4a3* expression and re-establishing the normal biochemical composition of the GBM of Col4a3^KO^ mice (Terada et al, 2002; Ambrosi & Rasmussen, 2005). Recent advances using CRISPR/Cas9 editing showed possible corrections in the mutations in *Col4a3* and *Col4a5* in podocytes collected from patient urine (Daga et al, 2020). Given previous reports that engraftment of human chorionic stem cells in Col4a3^KO^ mice can rescue the renal phenotype (Moschidou et al, 2016), our results with cord blood–derived MSCs offer a potential avenue for autologous cell-based therapies for patients with Alport syndrome.

Whereas horizontal gene transfer or cell fusion was captured in the kidneys of Col4a3^KO^ mice transplanted with NG2-cre transgenic donor bone marrow, the significance of mesangial cell/pericyte fusion is unknown. The contribution of mesangial cells to the GBM Col4a3 chain for functional recovery is less likely, given that these cells are not directly adjacent to the GBM. Our findings with MSC are also different from a previously report that used single injection of bone marrow–derived MSC to treat Col4a3^KO^ without efficacy (Prodromidi et al, 2006). This distinction is likely due to differences in the state and number of the MSCs used for treatment in irradiated mice (Prodromidi et al, 2006), and this warrants further study. New therapies for Alport syndrome are urgently needed (Chavez et al, 2022), and cell- or gene-based therapies hold the promise of correcting the genetic defects responsible for this devastating renal disease that impacts children.

## Materials and Methods

### Mice

Col4a3^KO^, R26StoplacZ^flox/flox^ (*lacZ* reporter), R26StopEYFP^flox/flox^ (YFP reporter), and NG2-Cre strains were previously described (Mao et al, 1999; Srinivas et al, 2001; LeBleu et al, 2009; LeBleu et al, 2013). CMV-Cre (stock 006054), Cdh5-Cre (stock 017968), Tie2-Cre (stock 008863), and R26StoptdTomato (tdTomato reporter, stock 007914) mice were purchased from Jackson Laboratories. Podocin-Cre mice were provided by Dr. Jordan Kreidberg, Children’s Hospital, Boston. Col4a3^L/L^ mice were generated by implantation of ESC harboring the Col4a3^tm1(EUCOMM)Ttsi^ allele and subsequent screening of progeny. The mice were bred with Rosa26-FLPe (stock 009086; Jackson Laboratories) to generate the conditional Col4a3 allele (Col4a3^L/L^). Adult female and male mice were used on C57BL/6 or C57BL/6 mixed backgrounds. All mice were housed under standard conditions at the Beth Israel Deaconess Medical Center or MD Anderson Cancer Center animal facility. All procedures were reviewed and approved by the Institutional Animal Care and Use Committee at Beth Israel Deaconess Medical Center or MD Anderson Cancer Center.

Bone marrow transplantation was performed as previously described (Sugimoto et al, 2006; LeBleu et al, 2009). Briefly, 5–8-wk-old mice were sub-lethally irradiated with 10 Gy of a ^137^cesium gamma source and were rescued by intravenous (i.v.) administration of 10^6^ unfractionated bone marrow cells via the retroorbital plexus 24 h after irradiation. Bone marrow cells were harvested aseptically from femur, tibia, and humerus of donor mice. Mice were euthanized for analysis 4–10 wk following bone marrow transplantation.

### mESC and iPSC culture and injection

mESC expressing GFP were a gift from Dr. George Daley, Children’s Hospital, Boston. iPSC (TTF1 line) were a gift from Dr. Konrad Hochedlinger, Massachusetts General Hospital, Boston. Undifferentiated mESC and iPSC were cultured on primary MEF feeder layer (Chemicon International) in DMEM (Gibco), supplemented with 15% FBS (Gibco), 1 M HEPES buffer (Sigma-Aldrich), 100 mM sodium pyruvate (Sigma-Aldrich), 0.12% monothioglycerol (Sigma-Aldrich), and 1.00 U/ml recombinant leukemia inhibitory factor (Chemicon). On the day of iPSC injection, feeder cells were removed by incubation of the cell suspension twice with PBS for 30 min at 37°C, and single cell iPSC, free from MEFs, as well as MEFs cultured without stem cells, were resuspended in PBS for injection into 8-wk-old Col4a3^KO^ mice (10^6^ cells in 100 μl PBS i.v. via the retroorbital plexus). The mice were euthanized at 14–15 wk of age. For differentiation, feeder cells were removed by incubation of the cell suspension twice with DMEM for 30 min at 37°C. mESC and iPSC were resuspended in culture medium lacking leukemia inhibitory factor. To induce embryoid body (EB) formation, iPSC were transferred to ultra-low attachment multiwell plates to allow their aggregation and prevent adherence to the plate. The EBs were cultured for 5 d and then dissociated with trypsin. Single cells were plated onto glass slide chambers or tissue culture dishes for five additional days. To transiently induce Cre expression in mESC and iPSC used in vitro, HSP-Cre-IRES-Luc plasmid (a gift from Dr. Jonathan Sleeman, KIT, Germany) was introduced using lipofectamine (Invitrogen) according to the manufacturer’s directions. For stable Cre expression in iPSC and in vivo studies, pBS598 EF1a-EGFPCre plasmid was used (plasmid 11923; Addgene [Le et al, 1999]).

### MSC culture and injection

Bone marrow cells were harvested by flushing the marrow from long bones of mice. The unfractionated bone marrow was incubated with ACK lysis buffer to deplete red blood cells from the cell preparation. The bone marrow cell preparation was then plated onto plastic tissue culture plates in DMEM medium supplemented with 10% FBS and 1X penicillin/streptomycin. Three days after plating, the medium was washed, removing all non-adhering cells, and the adhering mesenchymal cells were allowed to propagate to confluency. 0.5 to 1 × 10^6^ MSC were injected i.v. via the retroorbital plexus into non-irradiated 7–8-wk-old Col4a3^KO^ mice. The mice were then euthanized at 14–23 wk of age.

### Human cord blood MSC preparation

De-identified male human cord blood samples were kindly provided by Dr. Shahin Rafii (Weill Cornell Medical College) under institutional approval. Mononuclear cells were extracted as previously described (Newsome et al, 2003). The cells were plated and allowed to grow to confluency to enrich for MSC (CB-MSC). A preparation of 0.5 × 10^6^ cells in 100 μl PBS were injected systemically into Col4a3^KO^ mice. Control mice received 100 μl PBS systemically. The mice were aged up to 21 wk.

### Glomeruli co-culture studies

For glomeruli extraction, the mice were euthanized by cervical dislocation while under anesthesia. The chest cavity was opened, the heart exposed, and 30 ml of PBS containing magnetic beads (beads were purchased from Invitrogen, deactivated overnight in 0.2 M TRIS, pH 8.5, with 0.1% BSA, washed twice with PBS before dilution in 30 ml of PBS, 200 μl of beads were used per mouse) was perfused into the heart left ventricle at a rate of 5 ml per minute. Kidney blanching was observed nearly immediately following the beginning of perfusion (first 5 ml of perfusion) and indicated successful perfusion. The femoral vein was severed following blanching of the kidneys to allow perfusion of all 30 ml. The kidneys were then harvested, minced, and allowed to digest for 30 min in collagenase I/DNAseI mix (1 mg/ml collagenase I in PBS with 10 U/ml DNAse; using 2.5 ml for two kidneys) at 37°C, with intermittent vortexing every 15 min. The digested mixture was then filtered through a 100-μm-diameter pore cell strainer with an additional 10 ml of PBS, and the filtrate was centrifuged at room temperature at ~200*g* for 5 min. The supernatant was discarded, and the pellet resuspended into 5 ml of PBS and subjected to a magnet. The glomeruli with the trapped magnetic beads in the glomerular capillaries were successfully retained by the magnet while the remaining tissues were washed away three times with 5 ml of PBS. The glomeruli were cultured alone or in placed in co-culture with MSCs or iPSC with RPMI medium supplemented with 10% FBS and penicillin–streptomycin antibiotics at 37°C in 5% CO_2_. Glomerular outgrowth can be identified 1 wk after harvest.

For co-culture studies, ∼1,000 glomeruli (each glomeruli extraction from two adult mouse kidneys yields to approximately 5,000–10,000 glomeruli) were mixed with ∼2 million freshly extracted bone marrow MSC per two wells of a 12-well tissue culture dish in ∼3 ml of tissue culture medium. ∼1.5 to 3 million of iPSC or MSC were used in co-culture experiment with ∼1,000 glomeruli. These ratios of glomeruli to cells and tissue culture conditions were experimentally determined to allow for enough horizontal gene transfer or cellular fusion to be detected by PCR amplification of the recombined DNA. Lower cellular density (500 glomeruli with 500,000 bone marrow cells) and decreased cellular proximity (six-well tissue culture dish) also allowed for a detectable cellular fusion by PCR amplification of genomic DNA recombination, but at a lower efficiency. RPMI medium supplemented with 10% FBS and penicillin–streptomycin antibiotics was used unless otherwise indicated. When recombinant human TGFβ1 (10 ng/ml; Invitrogen) and anti-TGFβ1 neutralizing antibody (10 ng/ml; R&D Systems) were used, the glomeruli and bone marrow cells were co-cultured in RPMI medium supplemented with 0.01% FBS and penicillin–streptomycin antibiotics at 37°C in 5% CO_2_. The co-culture was allowed to incubate 24 h in RPMI medium supplemented with 0.01% FBS before addition of TGFβ1 or anti-TGFβ1 neutralizing antibody. The co-culture was then allowed to incubate for an additional 48 h. All other co-cultures were allowed to incubate from 24 to 72 h.

### LacZ substrate staining and YFP visualization

Kidneys and brains were embedded in OCT mounting medium and 5-μm frozen sections fixed at 4°C for 4 h in 4% PFA. Samples were washed three times with PBS, pH 7.3, and then stained overnight at 37°C with *lacZ* staining buffer (1 mg/ml 5-bromo-4-chloro-3-indolyl-beta-D-galactopyranoside [X-gal], 35 mM potassium ferrocyanide, 35 mM potassium ferricyanide, 2 mM magnesium chloride, 0.02% NP-40, and 0.01% sodium deoxycholate in PBS, pH 7.3). After washing with PBS, pH 7.3, the slides were counterstained with eosin. Mouse kidneys were also fixed in 4% PFA overnight at 4°C and equilibrated in 30% sucrose overnight at 4°C. The kidneys were then embedded in OCT compound. Frozen sections (5 μm) were mounted with VECTASHIELD Mounting Medium with DAPI (VECTASHIELD) and a glass coverslip, then visualized under a YFP fluorescent filter. Extracted glomeruli and glomerular outgrowth did not require any fixation, and YFP expression was visualized directly with a microscope with fluorescence capture capacity.

### Light microscopy and morphometric analyses

Kidneys were also fixed in formalin and paraffin sections used for H&E and PAS under standard conditions (Histology Core Facility, Beth Israel Deaconess Medical Center, Boston, MA, USA). H&E and sirius red (SR) staining of kidneys were also performed in the laboratory as previously described (Ozdemir et al, 2014). Morphometric analyses for the histological assessment of renal injury, here glomerular sclerosis and interstitial fibrosis, were performed as previously described (LeBleu et al, 2009).

### Renal function analyses

Creatinine concentrations were measured using the colorimetric assay Quantichrome (DICT-500) from BioAssays according to the manufacturer’s directions. Albumin concentrations were measured using the Mouse Albuminuria ELISA (Bethyl Laboratories) according to the manufacturer’s directions or using the colorimetric assay Quantichrome (DIAG-250) kit from BioAssays according to the manufacturer’s directions. Blood samples (100 μl) were collected, and plasma was separated from cellular components by centrifugation (~1500*g* for 5 min at room temperature) and stored frozen before analyses. BUN levels were measured using the colorimetric assay Quantichrome (DIUR-500) kit from BioAssays according to the manufacturer’s directions.

### RT–PCR and real-time RT–PCR analyses

Kidneys were homogenized in TRIzol (Invitrogen) and extracted according to the manufacturer’s directions. Synthesis of cDNA was performed using the Applied Biosystem cDNA synthesis kit according to the manufacturer’s directions. The following primers (and product size) were used for the RT–PCR.

**Table:** 

Gene	Primers	PCR product
*YFP*	5′-GCGACGTAAACGGCCACAAG-3′	600 bp
	5′-GCTTCTCGTTGGGGTCTTTGC-3′	
*Actb*	5′-CGTGGGCCGCCCTAGGCACCA-3′	200 bp
	5′-TTGGCCTTAGGGTTCAGGGGGG-3′	
*Col4a3*	5′-AAACGTGCACATGGACAAGA-3′	200 bp
	5′-CTCAGAGCCTGCACTTGTGA-3′	

### PCR amplification of recombined genomic DNA

Genomic DNA was extracted using DNA purification kit from QIAGEN according to the manufacturer’s direction. The PCR primer sequence and protocol for the detection of the recombined allele for YFP are as follows: Forward: 5′-AAGGGAGCTGCAGTGGAGTA-3′ with Reverse: 5′-GCCAGAGGCCACTTGTGTAG-3′ to amplify a 520-bp fragment for the unfloxed allele, and with Reverse: 5′-TGGTGCAGATGAACTTCAGG-3′ to amplify a 620-bp fragment for the floxed allele. Primer sequences for the internal control for genomic DNA amplification are as follows: Forward 5′-CTAGGCCACAGAATTGAAAGATCT-3′ and Reverse 5′-GTAGGTGGAAATTCTAGCATCATCC-3′ (320-bp product). The PCR primer sequences for the detection of the Col4a3 recombined allele are as follows: Del-Forward: 5′-AAGGCGCATAACGATACCAC-3′ and Del-Reverse: 5′-ACTGATGGCGAGCTCAGACC-3′.

### Protein purification of type IV collagen and Western blot analyses

Kidneys were harvested and homogenized in PBS with protease inhibitors (Roche) on ice. Samples were then incubated 2 h at 4°C in 1 M NaCl with 10 U/μl DNAse 1 (Invitrogen), 4 h at room temperature in 2% deoxycholate with protease inhibitors, washed with water, and incubated overnight with collagenase at 37°C (CLSAP; Worthington). ECM proteins in the supernatant were precipitated using 100% ethanol (1 h at 4°C) and denatured with SDS-sample buffer in boiling water. Denatured samples were separated on 8 or 10% SDS-polyacrylamide gels and blotted onto polyvinylidene fluoride membranes (Immobilon) by semi-dry method. The transferred protein was visualized with Coomassie brilliant blue. After blocking with TBS-T (TBS, 0.1% Tween 20) containing 5% non-fat milk, the membranes were incubated with anti-Col4a3 antibodies (rabbit anti-mouse Col4a3, a gift from Dr. Cosgrove, Boys Town National Research Center, Omaha, NE, 1:1,000 diluted) at 4°C overnight. The membranes were washed three times and incubated with 1:1,000 diluted HRP-conjugated anti-rabbit secondary antibody (Promega) at room temperature for 1 h. The immunoreactive bands were detected with an ECL detection system (Pierce Biotechnology).

### Immunolabeling of tissues and cells

Sagittal sections were embedded in OCT compound and snap frozen in liquid nitrogen. Thin frozen sections (5 μm) were fixed in ice-cold acetone for 20 min, blocked in 2% BSA, and immunostained with GFP antibody (used to detect YFP expression, ab290, 1:200 dilution; Abcam) or ß-galactosidase (A-11132, 1:200; Thermo Fisher Scientific). Cells growing on glass chambers were also fixed in ice-cold acetone for 20 min and blocked in 2% BSA. Nephrin and synaptopodin antibodies were a gift from Dr. Peter Mundel (Massachusetts General Hospital, Boston). DBA (Dolichos Biflorus Agglutinin, L32474; Thermo Fisher Scientific) and LTL (Lotus Tetragonolobus Lectin, L32480; Thermo Fisher Scientific) were used with FITC conjugation. All primary antibodies were diluted in dilution buffer (1% bovine serum albumin, 0.1% porcine skin gelatin, and 10 mM PBS, pH 7.2) at 1:200 dilution. TRITC- and FITC-conjugated secondary antibodies (Jackson Immunoresearch) were used at a dilution of 1:200 in PBS. The slides were mounted with VECTASHIELD Mounting Medium with DAPI (H1200; VECTASHIELD) and glass coverslip, and analyzed using an Axioskop 2 fluorescent microscope, AxioCam HRC camera and the Axiovision 4.3 software.

### Electron microscopy

For SEM, fixed samples containing 3% glutaraldehyde plus 2% paraformaldehyde in 0.1 M cacodylate buffer, pH 7.3, washed with 0.1 M cacodylate buffer, pH 7.3, post fixed with 1% cacodylate buffered osmium tetroxide, washed with 0.1 M cacodylate buffer, then in distilled water. Afterwards, the samples were sequentially treated with Millipore-filtered 1% aqueous tannic acid, washed in distilled water, treated with Millipore-filtered 1% aqueous uranyl acetate, and then rinsed thoroughly with distilled water. The samples were dehydrated with a graded series of increasing concentrations of ethanol, then transferred to a graded series of increasing concentrations of hexamethyldisilazane, and air dried overnight. Samples were mounted onto double-stick carbon tabs (Ted Pella. Inc.), which had been previously mounted onto glass microscope slides. The samples were then coated under vacuum using a Balzer MED 010 evaporator (Technotrade International) with platinum alloy for a thickness of 25 nm, then immediately flash carbon coated under vacuum. The samples were transferred to a desiccator for examination later. Samples were examined/imaged in a JSM-5900 scanning electron microscope (JEOL USA Inc.) at an accelerating voltage of 5 kV.

For TEM, samples were fixed with a solution containing 3% glutaraldehyde plus 2% paraformaldehyde in 0.1 M cacodylate buffer, pH 7.3, then washed in 0.1 M sodium cacodylate buffer and treated with 0.1% Millipore-filtered cacodylate buffered tannic acid, post-fixed with 1% buffered osmium tetroxide, and stained en bloc with 1% Millipore-filtered uranyl acetate. The samples were dehydrated in increasing concentrations of ethanol, infiltrated, and embedded in LX-112 medium. The samples were polymerized in a 60°C oven for ∼3 d. Ultrathin sections were cut in a Leica Ultracut microtome (Leica), stained with uranyl acetate and lead citrate, and examined in a JEM 1010 transmission electron microscope (JEOL USA Inc.) at an accelerating voltage of 80 kV. Digital images were obtained using AMT Imaging System (Advanced Microscopy Techniques Corp).

### Statistical analyses

Graphical representation of the data and statistical tests were performed using GraphPad Prism 9. The statistical test used for each data set is listed in the corresponding figure legends. The F-test was used to assess normality of distribution of samples. For two-groups comparison, unpaired *t* test with or without Welch’s correction was used for comparison of means. For multiple-groups comparison, one-way analysis of variance (ANOVA) with Dunnett’s multiple comparisons test or with Holm–Sidak’s multiple comparison test was used. The log-rank test was used to compare Kaplan–Meier survival curves. *P* values are reported as *P < 0.05, **P < 0.01, ***P < 0.001, ****P < 0.0001, *ns*: not significant.

## Supplementary Material

Reviewer comments
